# The complete chloroplast genome sequence of *Knema linifolia* (Myristicaceae)

**DOI:** 10.1080/23802359.2020.1791747

**Published:** 2020-07-22

**Authors:** Xiao-Qin Li, Feng-Liang Zhang, Chang-Li Mao, Tian Yang, Qi Zhao, Yu Wu

**Affiliations:** Yunnan Institute of Tropical Crops, Xishuangbanna, China

**Keywords:** *Knema linifolia*, chloroplast genome, Myristicaceae

## Abstract

*Knema linifolia* is a member of Myristicaceae. The *K. linifolia* chloroplast genome is found to be 155,754 bp in length and has a base composition of A (30.02%), G (19.30%), C (19.89%), and T (30.79%). The genome contained two short inverted repeat (IRa and IRb) regions (48,080 bp) which were separated by a large single copy (LSC) region (86,991 bp) and a small single copy (SSC) region (20,683 bp). The chloroplast genome has 89 protein-coding genes, 31 transfer RNA (tRNA) genes, and 8 ribosomal RNA (rRNA) genes. Further, complete chloroplast sequence of *K. linifolia* was aligned together with 2 species of Knema and 5 basal angiosperms species which have reported the complete chloroplast sequence. This complete chloroplast genome will provide valuable information for the development of DNA markers for future species resource development and phylogenetic analysis of *K. linifolia*.

*Knema linifolia*, belongs to Knema of Myristicaceae, is a tall arbor tree, and distributed from northeastern India through Bangladesh, Myanmar and Thailand to Indochina Peninsula (Editorial Committee of Chinese Academy of Sciences Flora 1979). So far, it has done little research in molecular biology, only as the taxonomic group with the other 10 species of Myristicaceae to discuss the taxonomic position of *Horsfieldia pandurifolia* (Wu et al. 2015). In this study, we characterized the complete chloroplast genome sequence of *K. linifolia* for phylogenetic analysis. The annotated genome sequence has been deposited Genbank under the accession number MN683753.

The fresh leaves of *K. linifolia* was collected in 2017 from Nangunhe River valley, Yunnan, China (99°04.39′E, 23°16.43′N), at the same time, we also took the seeds and brought them back to the base, its seedlings are planted and preserved in Yunnan Institute of Tropical Crops (YITC) and the number of voucher specimen is 20140475. The genome DNA of *K. linifolia* was extracted using the DNeasy Plant Mini Kit (QIAGEN, Valencia, CA), and its remaining DNA was stored in an ultra-low temperature freezer now. Genome sequencing was performed using Roche/454, sequencing libraries were prepared by the GS Titanium library preparation kit. The chloroplast genome assembled using CLC Genomic Workbench v3.6 (http://www.clcbio.com). The genes in the chloroplast genome were predicted using the DOGMA program (Wyman et al. 2004).

The circular genome is 155,754 bp in size, and comprises a large single copy (LSC) region (86,991 bp), a small single copy (SSC) region (20,683 bp), and two short inverted repeat (IRa and IRb) regions (48,080 bp). The base composition of the circular chloroplast genome is A (30.02%), G (19.30%), C (19.89%), and T (30.02%). The GC content of whole *K. linifolia* chloroplast genome was 39.19%. The chloroplast genome has 89 protein-coding genes, 31 transfer RNA (tRNA) genes, and 8 ribosomal RNA (rRNA) genes.

To study *K. linifolia* phylogenetic relationship with the angiosperms, *Horsfieldia pandurifolia* (Mao et al. 2019) and *Horsfieldia amygdalina* of Myristicaceae (Zhang et al. 2019) and other complete chloroplast genome sequences of angiosperms were download for analyses. The maximum likelihood phylogenetic was performed using MEGA X (Kumar et al. 2018) (Figure 1). A bootstrap analysis was performed on the resulting phylogenetic tree, and values were obtained after 1000 replications. The result shows that *K. linifolia* was clustered with other species and closely to *Horsfieldia pandurifolia* and *Horsfieldia amygdalina* chloroplast complete genome.

**Figure 1.**
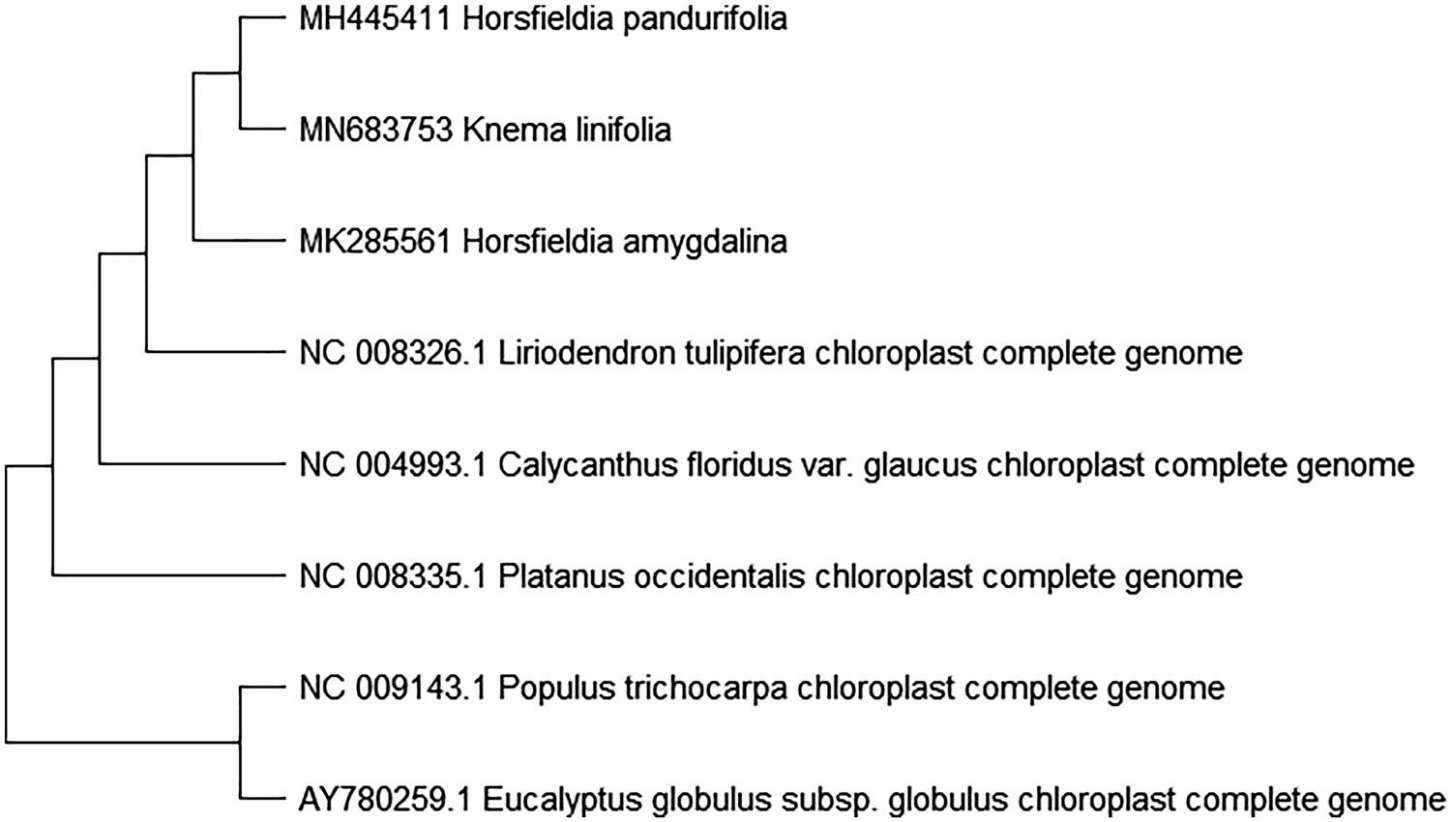
Maximum likelihood phylogenetic tree of *K. linifolia* with seven species based on complete chloroplast genome sequences. The gene’s accession number is list in figure and the data of *H. pandurifolia* and *H. amygdalina* come from author.

The complete chloroplast genome of *K. linifolia* would provide information on development of molecular markers and phylogenetic analysis in the future.

## Data Availability

The data that support the findings of this study are openly available in GenBank at https://www.ncbi.nlm.nih.gov/, reference number MN683753.
